# Crystal structure and Hirshfeld surface analysis of di­bromido­bis({(*S*)-2-[1-(di­methyl­amino)­eth­yl]phen­yl}di­phenyl­silanol-κ*O*)zinc(II)

**DOI:** 10.1107/S2056989025008989

**Published:** 2025-10-24

**Authors:** Julius Hättasch, Franziska Dorothea Klotz, Annika Schmidt, Carsten Strohmann

**Affiliations:** a Technische Universität Dortmund, Fakultät für Chemie und Chemische Biologie, Otto-Hahn-Strasse 6, 44227 Dortmund, Germany; National Taras Shevchenko University of Kyiv, Ukraine

**Keywords:** crystal structure, zinc bromide complex, chiral silanol ligand, Hirshfeld surface analysis, zwitterionic structure, crystal voids

## Abstract

Mol­ecules of the title compound, [ZnBr_2_(C_22_H_25_NOSi)_2_], are situated across a twofold axis passing through the central Zn atom in the uncommon space group C222_1_. The organic ligands feature migration of Si—OH protons to the amine substituents, while the resulting zwitterions adopt strong charge-assisted intra­molecular N—H⋯O hydrogen bonds [N⋯O = 2.5895 (13) Å]. They act as monodentate ligands through highly nucleophilic silanolate O atoms, completing a distorted ZnBr_2_O_2_ tetra­hedral environment of the metal ions [Zn—O = 1.9509 (9) Å]. In the crystal, weak C—H⋯Br hydrogen bonds govern the assembly of a two-dimensional pattern parallel to the (110) plane, further consolidated by C—H⋯π bonding. These findings are in line with the results of Hirshfeld surface analysis. The latter reveals the prevalence of H⋯H inter­actions (67.9%), which are followed by H⋯C/C⋯H (21.5%) and Br⋯H/H⋯Br (10.5%) contacts. The fingerprint plots also indicate the presence of crystal voids that are consistent with a relatively loose packing.

## Abstract 2

Monodentate zwitterionic silanolate (sil) ligands in [ZnBr_2_(sil)_2_] coordinate to zinc(II) centres, yielding complexes with intra­molecular N—H⋯O hydrogen bonds and distorted ZnBr_2_O_2_ tetra­hedral environments that assemble into loosely packed two-dimensional layers stabilized by weak inter­molecular C—H⋯Br inter­actions.

## 1. Chemical context

Silanols are a versatile class of compounds that are used both in the chemical industry and in organic synthesis. Their importance ranges from their use as column materials, for example, in the form of silica gel (Ritgen, 2019 ▸), to their role as reactants in coupling reactions (Hirabayashi *et al.*, 1998 ▸). In addition, silanol functionalities have been employed as bio­iso­steres for hydroxyl groups in drug development, both by modifying known compounds and by designing new silanol-based mol­ecules. Several of these derivatives have shown promising biological properties (Showell *et al.*, 2006 ▸; Tacke *et al.*, 1989 ▸, 1991 ▸). Silanols can also be used as temporary ligands to control the regioselectivity of metal-catalyzed reactions (Yamagishi *et al.*, 2023 ▸). Furthermore, zinc siloxides can act as protected forms of silanols, bypassing the often poor stability of silanols towards condensation (Golz *et al.*, 2017 ▸). The work presented here shows a stable and carbon-chiral silanol mol­ecule (**1**), which can be used for the synthesis of transition-metal complexes.
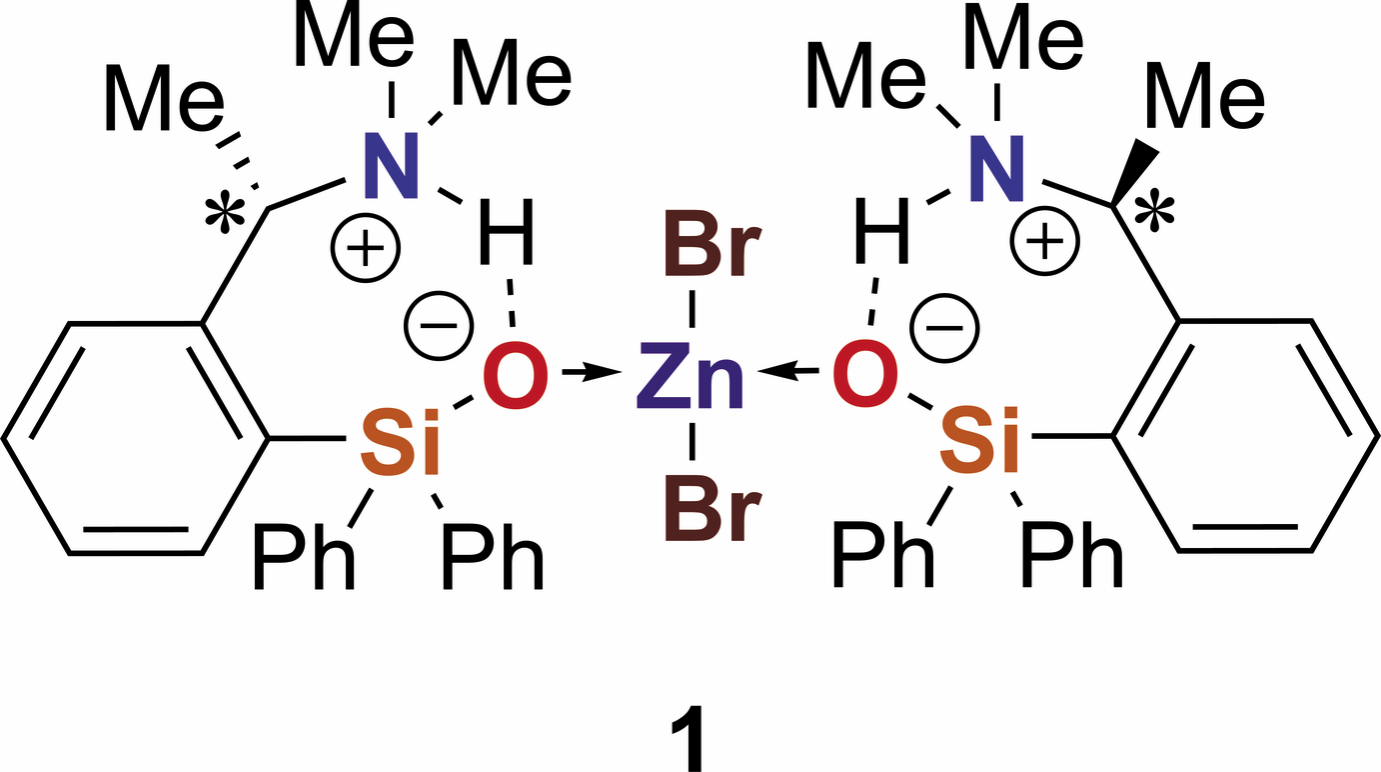


In the present work, we report on the tetra­hedral zinc bromide complex di­bromido­bis({(*S*)-2-[1-(di­methyl­amino)­eth­yl]phen­yl}di­phenyl­silanol-κ*O*)zinc(II), ZnBr_2_*L*_2_ (**1**), adopted by the enanti­omerically pure {(*S*)-2-[1-(di­methyl­amino)­eth­yl]phen­yl}di­phenyl­silanol (**2**) and how the desired silanolate structure (Si—O^−^) may be generated by intra­molecular prototropic migration involving silanol (Si—OH group) and the tertiary amino groups (Scheme 2). The highly nucleophilic silanolate O-donor sites are prone to coordination to the Lewis acid Zn^2+^, while retaining markedly strong inter­action with the H atoms, which are now located at the adjacent N-atom sites.
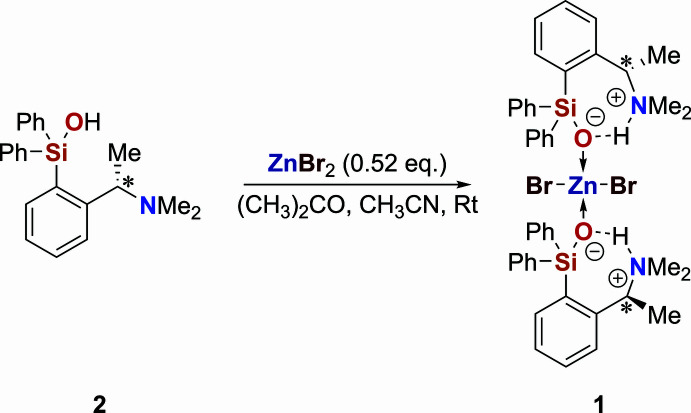


## 2. Structural commentary

**Figure 1:**
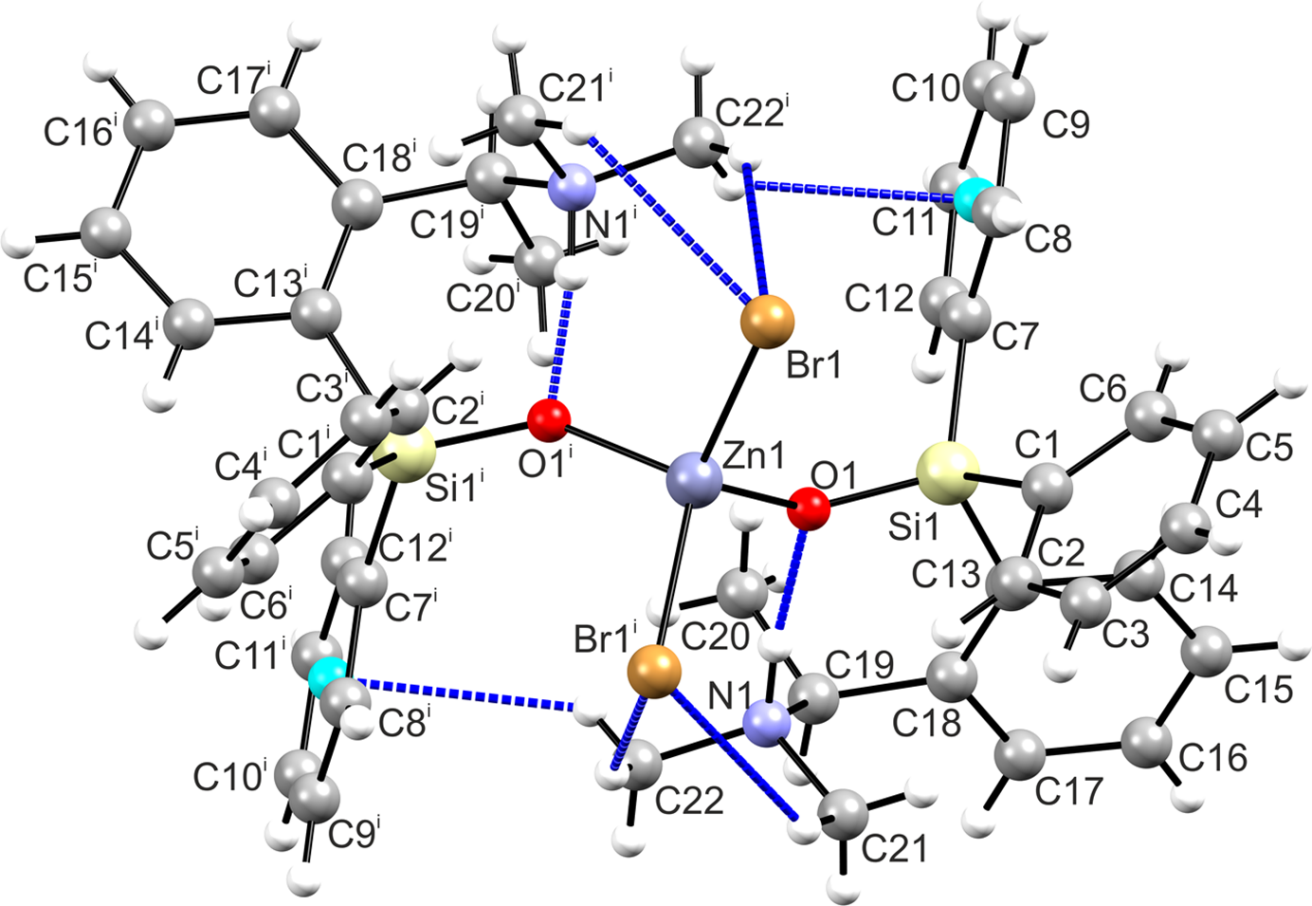
Mol­ecular structure of **1**, drawn with 50% probability displacement ellipsoids, showing intramolecular interactions (dashed blue lines). Relevant ring centroids are represented by light-blue spheres. [Symmetry code: (i) *x*, −*y* + 1, −*z* + 1.]

The title complex, **1**, crystallizes at room temperature from aceto­nitrile solution as a mol­ecular complex (Fig. 1 ▸ and Table 1 ▸). The asymmetric part of the structure comprises one half of a mol­ecule situated across a twofold axis passing through the Zn1 atom. All bond lengths and angles are within the expected ranges as found in the Cambridge Structural Database (CSD; Groom *et al.*, 2016 ▸; WebCSD September 2025). The zinc ion adopts a slightly distorted tetra­hedral coordination geometry, with the largest angle at the central atom sustained with the two bromide ligands [114.035 (11)°] and the smallest angle with the two silanolate O atoms [102.34 (6)°]. The absolute configuration at the stereogenic centre was confirmed as *S* by X-ray diffraction. The refined Flack *x* parameter of −0.0041 (15) supports the assignment and matches the configuration of the chiral precursor.

The organic ligand exists in a conformation that is favourable for intra­molecular hydrogen bonding involving the precisely positioned silanol and amino groups. Previous X-ray structure studies revealed similar geometry for the non-coordinated species, with the H atom located at the O atom of the silanol group (Langenohl, 2021 ▸). In the present case, the coordination to the zinc ion enhances the acidity of the silanol group and promotes the proton transfer to the amine group, thus generating a zwitterionic species in the crystal structure of the complex. With this proton transfer, the charge-assisted intra­molecular N^+^—H⋯O^−^ hydrogen bond actualizes over the neutral reverse pattern O—H⋯N seen in the non-coordinated precursor **2**. Table 2 ▸ compares the observed hydrogen-bond geometries in **1** with those in **2** (Langenohl, 2021 ▸) and two related structures from the CSD. The crystals reported by Robert and co-workers (refcode BAYVAB; Nguyen *et al.*, 2017 ▸) and by Wang (KANNUK; Wang, 2011 ▸) feature the best comparable inter­molecular N^+^—H⋯O^−^⋯Zn motif. Within this comparison, the present hydrogen bond appears to be the strongest, with the shortest donor–acceptor distance and the hydrogen-bond angle nearest to 180°.

The mol­ecular structure of the complex is additionally consolidated by a set of weaker inter­actions, which include symmetry-related pairs of two C—H⋯Br bonds [C⋯Br = 3.7373 (14) and 3.8552 (14) Å] and C—H⋯π bonds with methyl donors (Table 3 ▸). Also, one can identify a tetrel bond between Br1 and Si1. It is characterized by a Br1⋯Si1 distance of 4.1850 (6) Å and a Br1⋯Si1—C13 angle of 163.03 (4)°. This inter­action is likely responsible for the elongation of the Si1—C13 bond by 0.018 (3) Å with respect to the other two Si—C bonds (see Table 1 ▸).

## 3. Supra­molecular features

**Figure 2:**
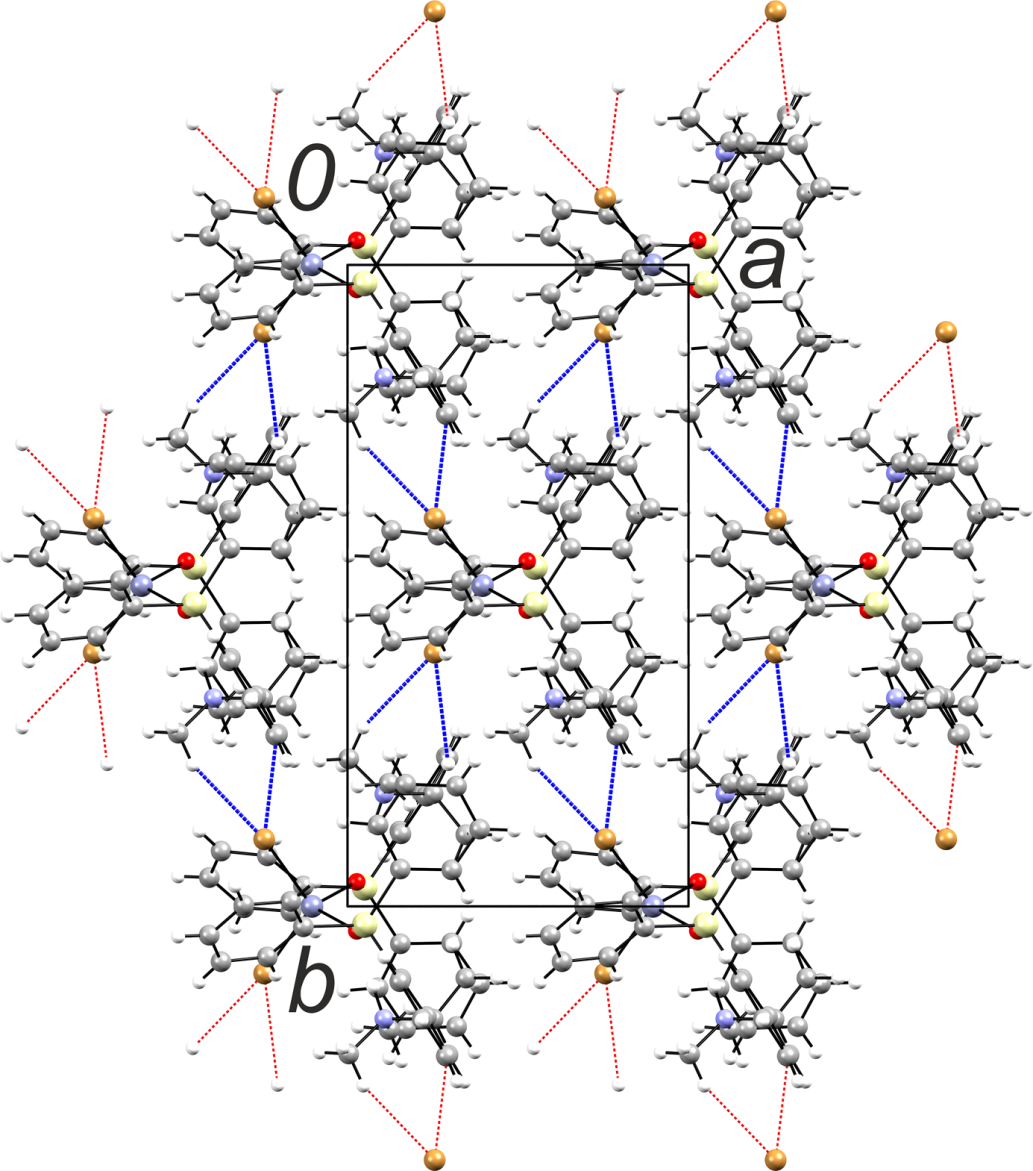
The mol­ecular packing of **1**, viewed along [001], with the unit cell shown as a black outline. The most significant inter­molecular inter­actions within the layer are represented by C—H⋯Br hydrogen bonds (blue dashed lines).

Being dominated rather by dispersion forces, the crystal packing is relatively loose, with a packing index of 67.9. Few identified inter­molecular inter­actions are represented by very weak hydrogen bonding (Table 3 ▸). The mol­ecules are linked by double hydrogen bonds C19—H⋯Br1^ii^ and C21—H⋯Br1^ii^ [symmetry code: (ii) *x* + 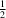, *y* − 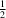, *z*], which produces a two-dimensional network parallel to (110) (Fig. 2 ▸). These layers face each other with phenyl groups and are linked by mutual C—H⋯π bonds, namely, C15—H⋯*Cg*(C1–C6)^iii^ [*Cg* is the group centroid; symmetry code: (iii) −*x* + 1, *y*, −*z* + 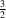]. One can note that beyond the above strongest intra­molecular hydrogen bond, five out of six C—H⋯Br and C—H⋯π inter­actions are generated with aliphatic C—H donors in the α-position relative to the N—H^+^ site.

**Figure 3:**
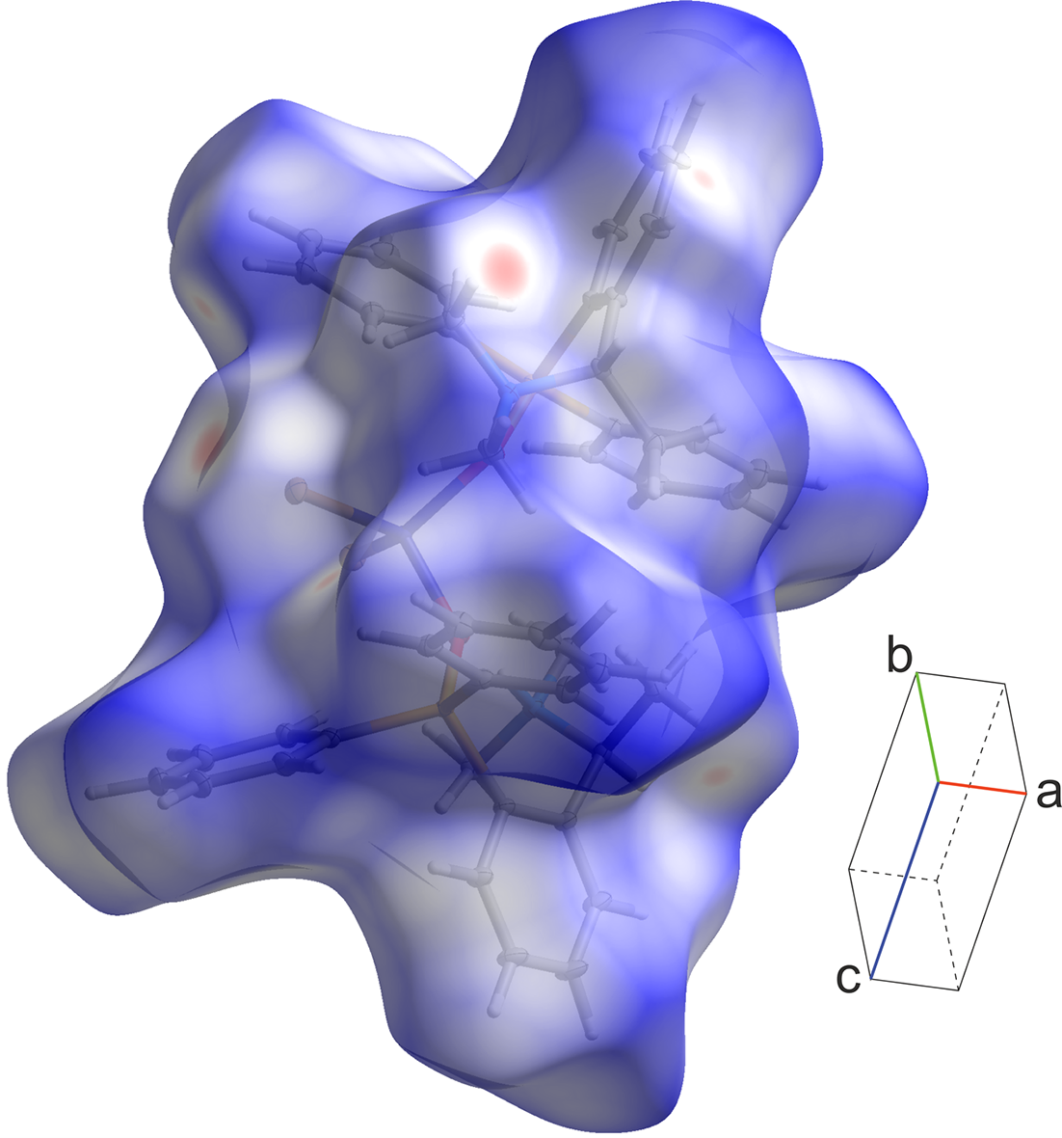
Three-dimensional Hirshfeld surface analysis of **1** mapped over *d*_norm_. The red regions correspond to specific inter­molecular contacts.

In order to better understand the inter­molecular inter­actions, a Hirshfeld surface analysis was carried out. The Hirshfeld surface and fingerprint plots (McKinnon *et al.*, 2007 ▸) were created using *CrystalExplorer21* (Spackman *et al.*, 2021 ▸). The Hirshfeld surface shown in Fig. 3 ▸ was mapped over *d*_norm_ in the range from −0.143 to 1.476 a.u. It highlights 12 close contacts as red spots, whereas blue regions indicate inter­molecular separations above the sum of the atomic radii. The most intense spots correspond to the Br⋯H/H⋯Br inter­actions described above, although even in such cases the contacts approach the normal van der Waals (vdW) separations. Each of the two silanolate ligands contributes six contacts, which complete the mol­ecular environments in the layers parallel to the (110) plane.

**Figure 4:**
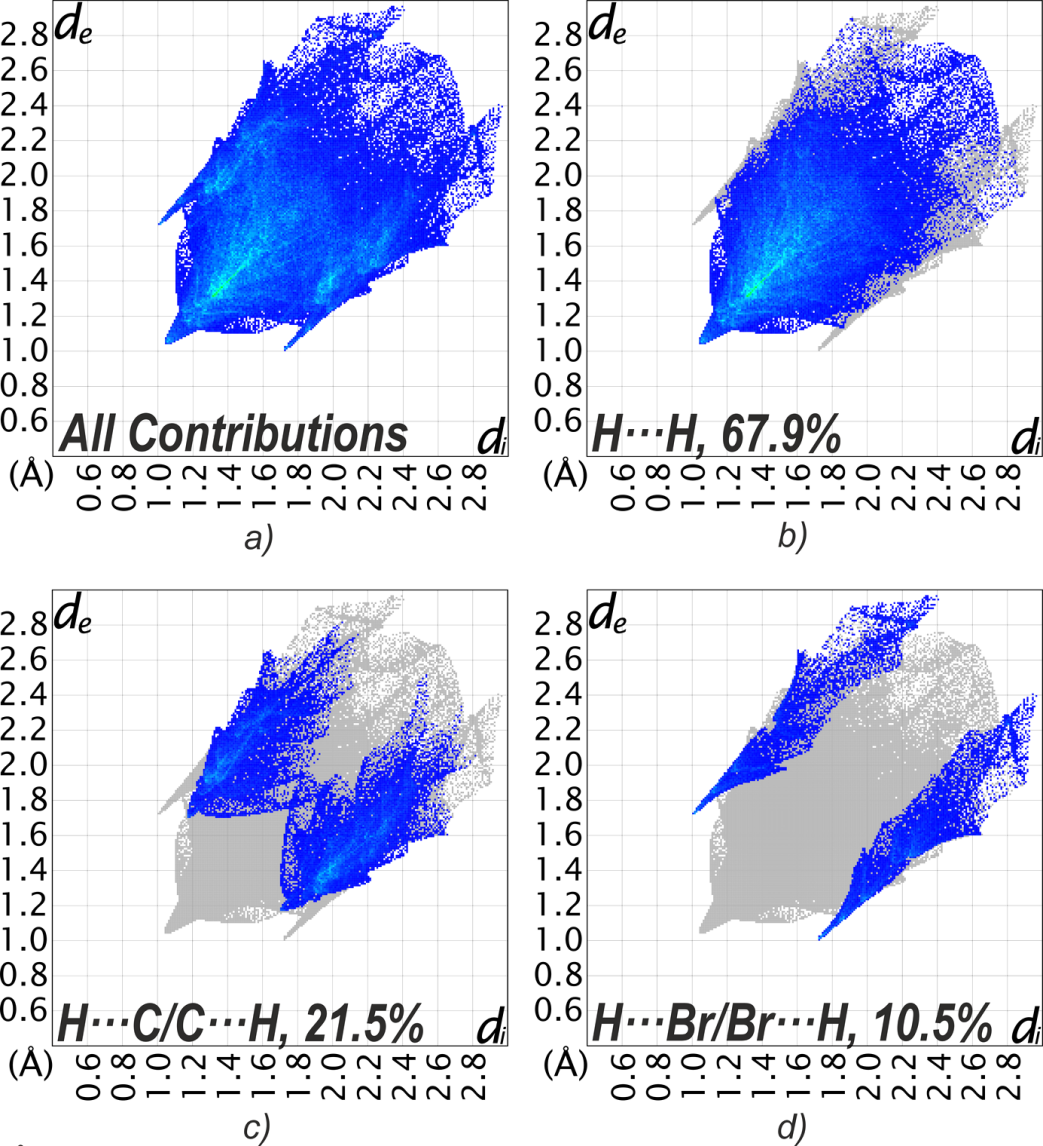
Two-dimensional fingerprint plots for **1**, showing (*a*) all and (*b*)–(*d*) selected inter­actions in the crystal. *d*_e_ and *d*_i_ represent the distances from a point on the Hirshfeld surface to the nearest external or inter­nal atom, respectively.

**Figure 5:**
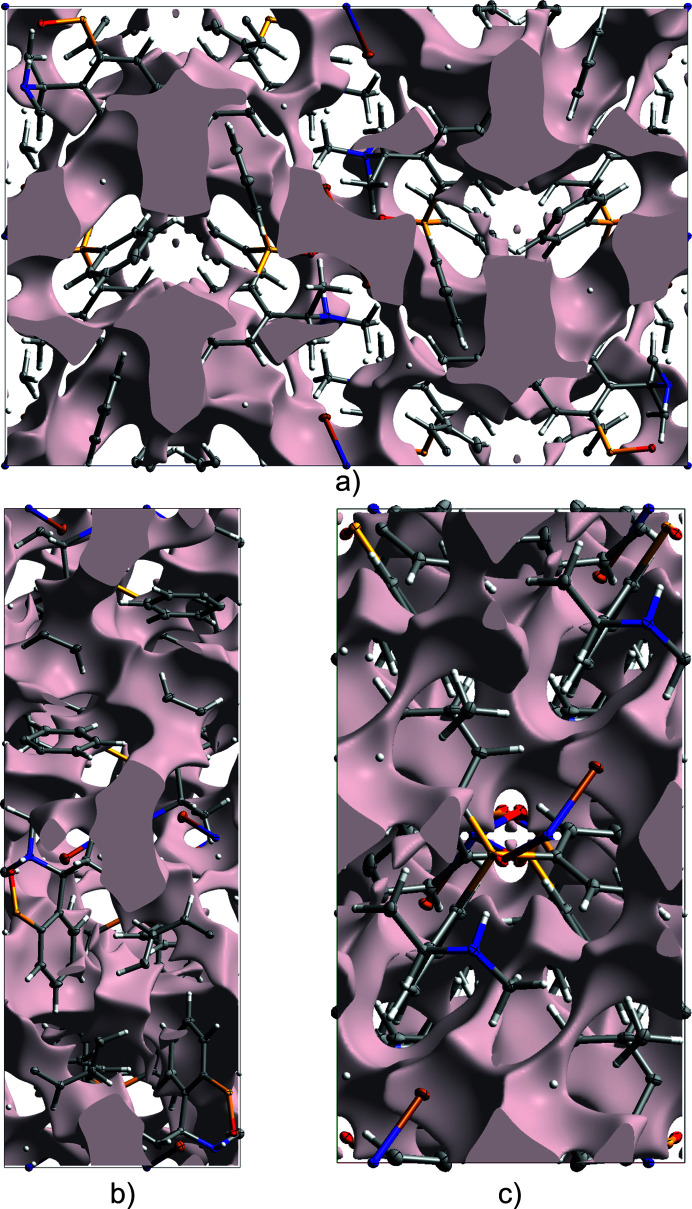
Views of the calculated *Crystal Voids* isosurface in the (*a*) [100], (*b*) [010] and (*c*) [001] direction.

The relative contributions of the inter­molecular inter­actions in compound **1** were also analyzed using two-dimensional fingerprint plots (Spackman & McKinnon, 2002 ▸). As expected, H⋯H contacts are the most significant, accounting for 67.9% of the surface (Fig. 4 ▸), reflecting the importance of vdW inter­actions to the packing. H⋯C/C⋯H contacts contribute 21.5%, which is consistent with the presence of weak C—H⋯π inter­actions or general hydro­phobic contacts involving the phenyl substituents. The Br⋯H/H⋯Br inter­actions account for 10.5% of the surface area and represent the main directional contacts in the supra­molecular structure. There are no C⋯C contacts, in accordance with the absence of significant π–π inter­actions. The fingerprint plots also show a diffuse collection of points above *d*_e_, *d*_i_ = 2.5 Å, indicating voids in the crystal structure. This was further investigated by calculating the *Crystal Voids* isosurface (Turner *et al.*, 2011 ▸) in *CrystalExplorer21* for the whole unit cell with the isovalue set to 0.002 e au^−3^. The calculated surface with the rescale surface property set to −0.005 to 0.000 is depicted in Fig. 5 ▸. For the calculation of the void volume, capping faces are generated on the boundary of the unit cell. With 13.3% (561 Å^3^) of the volume outside of the isosurface, the crystal of **1** appears packed loose.

## 4. Database survey

A search of the Cambridge Structural Database (Groom *et al.*, 2016 ▸; WebCSD July 2025) for zinc bromide complexes coordinated by sil­oxy groups revealed several structures, four of which contained bridged Zn—O bonds in which one O atom coordinates to two zinc centres. They are tetra­bromo­dizinc(II) complexes involving μ_2_-1-{[(­oxy)(diphen­yl)sil­yl]meth­yl}pi­per­i­dinium, μ_2_-1-{[­oxy(dimeth­yl)sil­yl]meth­yl}-5-methyl­py­r­rol­i­din-1-ium and μ_2_-1-{[(­oxy)(dimeth­yl)sil­yl]meth­yl}-2,6-di­methyl­pi­per­i­dinium (CSD refcodes VUPFAO, VUPFES and VUPFIW, respectively; Däschlein & Strohmann, 2009 ▸), as well as bis­[μ_2_-(1-{[(hy­droxy)(dimeth­yl)sil­yl]meth­yl}piperidiniumato)]tetra­bromo­dizinc(II) (WUDPAN; Däschlein *et al.*, 2009 ▸). A closely related structure differs only by the replacement of one phenyl group with a methyl group. The structure of di­bromo­bis­({2-[1-(di­methyl­amino)­eth­yl]phen­yl}(meth­yl)phenyl­silanol)zinc(II) acetone solvate (IFUSEL; Langenohl *et al.*, 2023 ▸) shows a tetra­hedrally coordinated zinc centre, which accommodates two silanolate O ligands. In this complex, the proton is also transferred from the silanol group to the amino group, resulting in a zwitterionic species, as in the present structure. This transfer is also likely induced by an increase in the acidity of the silanol O atom upon coordination to the Zn^2+^ ion.

## 5. Synthesis and crystallization

The synthesis of the carbon-chiral precursor {(*S*)-2-[1-(di­methyl­amino)­eth­yl]phen­yl}di­phenyl­silanol (**2**) was conducted according to a previously established procedure (Langenohl, 2021 ▸). For the synthesis of the title compound (**1**), **2** (347.53 g mol^−1^, 60.0 mg, 0.17 mmol, 1.00 equiv.) and zinc bromide (225.19 g mol^−1^, 20.2 mg, 0.09 mmol, 0.52 equiv.) were each dissolved in 2 ml acetone. The ligand solution was dripped into the metal bromide solution and the solvent was evaporated slowly at room temperature. The resulting solid was not suitable for single-crystal X-ray diffraction and was therefore dissolved in aceto­nitrile and the solvent evaporated slowly at room temperature again. After 4 d, the product was obtained as colourless blocks.

## 6. Refinement

Crystal data, data collection and structure refinement details are summarized in Table 4 ▸. All H atoms were located in difference maps and then refined with isotropic displacement parameters.

## Supplementary Material

Crystal structure: contains datablock(s) I. DOI: 10.1107/S2056989025008989/nu2013sup1.cif

Structure factors: contains datablock(s) I. DOI: 10.1107/S2056989025008989/nu2013Isup2.hkl

CCDC reference: 2495680

Additional supporting information:  crystallographic information; 3D view; checkCIF report

## Figures and Tables

**Table 1:** Selected geometrical parameters (Å, °) for **1**

Zn1—O1	1.9509 (9)	Si1—C1	1.8802 (13)
Zn1—Br1	2.39687 (17)	Si1—C7	1.8820 (13)
Si1—O1	1.6135 (9)	Si1—C13	1.8996 (13)
			
Br1—Zn1—Br1^i^	114.035 (11)	O1^i^—Zn1—O1	102.34 (6)
O1—Zn1—Br1	108.75 (3)	Si1—O1—Zn1	137.64 (5)
O1^i^—Zn1—Br1^i^	111.18 (3)		

Symmetry code: (i) *x*, −*y* + 1, −*z* + 1.

**Table 2:** Hydrogen-bond geometry (Å, °) for **1** compared with other hydrogen-bond geometries

	*D*—H⋯*A*	*D*—H	H⋯*A*	*D*⋯*A*	*D*—H⋯*A*
**1**	N1—H1⋯O1	0.95 (2)	1.65 (2)	2.5895 (13)	172 (2)
(Langenohl, 2021 ▸)	O1—H1⋯N1	0.840 (2)	1.795 (2)	2.628 (2)	171.48 (11)
	O2—H2⋯N2	0.840 (2)	1.816 (2)	2.637 (2)	165.20 (11)
BAYVAB	N6—H6⋯O4	0.89 (8)	1.77 (7)	2.634 (7)	163 (7)
	N4—H4⋯O1	0.99 (10)	1.68 (11)	2.637 (10)	163 (7)
KANNUK	N2—H2⋯O3	0.90 (1)	1.82 (5)	2.718 (8)	169 (7)
	N7—H7⋯O7	0.90 (1)	1.84 (3)	2.716 (8)	163 (9)

Note: Bond lengths and angles involving H atoms in the literature structures should be inter­preted with care, since the H-atom positions were geometrically constrained during refinement. In contrast, all H atoms in the present structure were refined freely.

**Table 3:** Hydrogen-bond geometry (Å, °)

*D*—H⋯*A*	*D*—H	H⋯*A*	*D*⋯*A*	*D*—H⋯*A*
C21—H21*A*⋯Br1^i^	0.96 (2)	3.10 (2)	3.8552 (14)	137.0 (18)
C22—H22*A*⋯Br1^i^	0.94 (2)	2.95 (2)	3.7373 (14)	142.1 (17)
C19—H19⋯Br1^ii^	0.92 (2)	3.09 (2)	3.8944 (11)	146.9 (17)
C21—H21*C*⋯Br1^ii^	0.95 (3)	2.86 (3)	3.7615 (14)	159 (2)
C22—H22*B*⋯*Cg*(C7–C12)^i^	0.90 (2)	2.84 (2)	3.4972 (15)	131 (2)
C15—H15⋯*Cg*(C1–C6)^iii^	0.97 (2)	3.11 (2)	3.7553 (16)	125 (2)

Symmetry codes: (i) 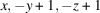; (ii) 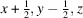; (iii) 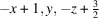. *Cg* is the group centroid.

**Table 4:** Experimental details

Crystal data	
Chemical formula	[ZnBr_2_(C_22_H_25_NOSi)_2_]
*M*_r_	920.23
Crystal system, space group	Orthorhombic, *C*222_1_
Temperature (K)	100
*a*, *b*, *c* (Å)	9.3111 (4), 17.5025 (8), 26.0142 (12)
*V* (Å^3^)	4239.5 (3)
*Z*	4
Radiation type	Mo *K*α
μ (mm^−1^)	2.56
Crystal size (mm)	0.37 × 0.35 × 0.27

Data collection	
Diffractometer	Bruker D8 VENTURE area detector
Absorption correction	Multi-scan (*SADABS*; Bruker, 2016 ▸)
*T*_min_, *T*_max_	0.476, 0.568
No. of measured, independent and observed [*I* > 2σ(*I*)] reflections	242533, 10338, 9837
*R*_int_	0.058
(sin θ/λ)_max_ (Å^−1^)	0.834

Refinement	
*R*[*F*^2^ > 2σ(*F*^2^)], *wR*(*F*^2^), *S*	0.018, 0.046, 1.06
No. of reflections	10338
No. of parameters	340
H-atom treatment	All H-atom parameters refined
Δρ_max_, Δρ_min_ (e Å^−3^)	0.39, −0.35
Absolute structure	Flack *x* determined using 4306 quotients [(*I*^+^−(*I*^−^)]/[(*I*^+^)+(*I*^−^)] (Parsons *et al.*, 2013 ▸)
Absolute structure parameter	−0.0041 (15)

Computer programs: *SAINT* (Bruker, 2016 ▸), *SHELXT* (Sheldrick, 2015 ▸), *SHELXL* (Sheldrick, 2008 ▸) and *OLEX2* (Dolomanov *et al.*, 2009 ▸).

## supplementary crystallographic information

### Dibromidobis({(S)-2-[1-(dimethylamino)ethyl]phenyl}diphenylsilanol-κO)zinc(II) Crystal data

**Table:**

[ZnBr_2_(C_22_H_25_NOSi)_2_]	*D*_x_ = 1.442 Mg m^−^^3^
*M**_r_* = 920.23	Mo *K*α radiation, λ = 0.71073 Å
Orthorhombic, *C*222_1_	Cell parameters from 9405 reflections
*a* = 9.3111 (4) Å	θ = 2.5–35.9°
*b* = 17.5025 (8) Å	µ = 2.56 mm^−^^1^
*c* = 26.0142 (12) Å	*T* = 100 K
*V* = 4239.5 (3) Å^3^	Block, colourless
*Z* = 4	0.37 × 0.35 × 0.27 mm
*F*(000) = 1888	

### Dibromidobis({(S)-2-[1-(dimethylamino)ethyl]phenyl}diphenylsilanol-κO)zinc(II) Data collection

**Table:**

Bruker D8 VENTURE area detector diffractometer	10338 independent reflections
Radiation source: microfocus sealed X-ray tube, Incoatec Iµs	9837 reflections with *I* > 2σ(*I*)
HELIOS mirror optics monochromator	*R*_int_ = 0.058
Detector resolution: 10.4167 pixels mm^-1^	θ_max_ = 36.4°, θ_min_ = 2.3°
ω and φ scans	*h* = −15→15
Absorption correction: multi-scan (SADABS; Bruker, 2016)	*k* = −29→29
*T*_min_ = 0.476, *T*_max_ = 0.568	*l* = −43→43
242533 measured reflections	

### Dibromidobis({(S)-2-[1-(dimethylamino)ethyl]phenyl}diphenylsilanol-κO)zinc(II) Refinement

**Table:**

Refinement on *F*^2^	Hydrogen site location: difference Fourier map
Least-squares matrix: full	All H-atom parameters refined
*R*[*F*^2^ > 2σ(*F*^2^)] = 0.018	*w* = 1/[σ^2^(*F*_o_^2^) + (0.0262*P*)^2^ + 0.5577*P*] where *P* = (*F*_o_^2^ + 2*F*_c_^2^)/3
*wR*(*F*^2^) = 0.046	(Δ/σ)_max_ = 0.003
*S* = 1.06	Δρ_max_ = 0.39 e Å^−^^3^
10338 reflections	Δρ_min_ = −0.35 e Å^−^^3^
340 parameters	Absolute structure: Flack *x* determined using 4306 quotients [(I+)-(I-)]/[(I+)+(I-)] (Parsons *et al.*, 2013)
0 restraints	Absolute structure parameter: −0.0041 (15)
Primary atom site location: dual	

### Dibromidobis({(S)-2-[1-(dimethylamino)ethyl]phenyl}diphenylsilanol-κO)zinc(II) Special details

**Table:**

Geometry. All esds (except the esd in the dihedral angle between two l.s. planes) are estimated using the full covariance matrix. The cell esds are taken into account individually in the estimation of esds in distances, angles and torsion angles; correlations between esds in cell parameters are only used when they are defined by crystal symmetry. An approximate (isotropic) treatment of cell esds is used for estimating esds involving l.s. planes.

### Dibromidobis({(S)-2-[1-(dimethylamino)ethyl]phenyl}diphenylsilanol-κO)zinc(II) Fractional atomic coordinates and isotropic or equivalent isotropic displacement parameters (Å^2^)

**Table:**

	*x*	*y*	*z*	*U*_iso_*/*U*_eq_	
Br1	0.25556 (2)	0.60464 (2)	0.53188 (2)	0.01751 (3)	
Zn1	0.39569 (2)	0.500000	0.500000	0.00923 (3)	
Si1	0.55007 (4)	0.47098 (2)	0.61362 (2)	0.01029 (5)	
O1	0.52706 (10)	0.46179 (5)	0.55246 (3)	0.01189 (14)	
N1	0.60742 (12)	0.32472 (6)	0.52845 (4)	0.01226 (16)	
H1	0.575 (3)	0.3754 (13)	0.5341 (9)	0.022 (5)*	
C1	0.37053 (13)	0.46827 (8)	0.64665 (5)	0.01371 (19)	
C2	0.26485 (15)	0.42116 (8)	0.62474 (5)	0.0176 (2)	
H2	0.289 (3)	0.3938 (14)	0.5950 (9)	0.025 (6)*	
C3	0.12921 (16)	0.41381 (11)	0.64674 (6)	0.0234 (3)	
H3	0.065 (3)	0.3821 (15)	0.6304 (10)	0.032 (6)*	
C4	0.09460 (17)	0.45510 (11)	0.69067 (6)	0.0261 (3)	
H4	0.003 (3)	0.4532 (16)	0.7029 (10)	0.036 (7)*	
C5	0.19603 (17)	0.50221 (11)	0.71296 (5)	0.0247 (3)	
H5	0.170 (3)	0.5321 (16)	0.7450 (10)	0.040 (7)*	
C6	0.33367 (16)	0.50790 (9)	0.69169 (5)	0.0185 (2)	
H6	0.405 (3)	0.5389 (14)	0.7069 (9)	0.025 (6)*	
C7	0.65495 (14)	0.55833 (7)	0.63287 (5)	0.01323 (19)	
C8	0.58967 (16)	0.62692 (7)	0.64829 (5)	0.0163 (2)	
H8	0.488 (3)	0.6317 (14)	0.6486 (9)	0.024 (6)*	
C9	0.67035 (19)	0.69061 (8)	0.66218 (6)	0.0203 (3)	
H9	0.622 (3)	0.7365 (16)	0.6725 (11)	0.030 (6)*	
C10	0.81991 (19)	0.68746 (9)	0.66063 (6)	0.0226 (3)	
H10	0.873 (3)	0.7272 (16)	0.6686 (10)	0.029 (6)*	
C11	0.88751 (18)	0.62054 (9)	0.64493 (6)	0.0226 (3)	
H11	0.989 (3)	0.6187 (15)	0.6434 (9)	0.027 (6)*	
C12	0.80564 (15)	0.55698 (8)	0.63162 (6)	0.0176 (2)	
H12	0.852 (2)	0.5143 (14)	0.6239 (9)	0.024 (6)*	
C13	0.65665 (13)	0.38691 (7)	0.63965 (4)	0.01183 (18)	
C14	0.65333 (16)	0.38275 (8)	0.69366 (5)	0.0163 (2)	
H14	0.608 (3)	0.4211 (13)	0.7111 (8)	0.019 (5)*	
C15	0.71940 (18)	0.32442 (9)	0.72139 (5)	0.0209 (3)	
H15	0.717 (2)	0.3224 (12)	0.7586 (8)	0.020 (5)*	
C16	0.78960 (19)	0.26659 (9)	0.69506 (6)	0.0233 (3)	
H16	0.821 (3)	0.2275 (16)	0.7113 (10)	0.033 (7)*	
C17	0.79479 (17)	0.26874 (8)	0.64160 (5)	0.0195 (2)	
H17	0.842 (3)	0.2269 (15)	0.6232 (10)	0.028 (6)*	
C18	0.73154 (13)	0.32825 (7)	0.61381 (4)	0.01269 (18)	
C19	0.75106 (15)	0.32433 (6)	0.55560 (4)	0.01300 (17)	
H19	0.795 (2)	0.2781 (13)	0.5488 (8)	0.019 (5)*	
C20	0.84696 (14)	0.38824 (8)	0.53486 (5)	0.0171 (2)	
H20A	0.939 (3)	0.3884 (15)	0.5554 (10)	0.029 (6)*	
H20B	0.808 (2)	0.4402 (13)	0.5386 (8)	0.019 (5)*	
H20C	0.872 (3)	0.3816 (15)	0.5020 (11)	0.034 (6)*	
C21	0.50345 (15)	0.26999 (8)	0.55163 (5)	0.0162 (2)	
H21A	0.418 (3)	0.2724 (14)	0.5309 (9)	0.024 (6)*	
H21B	0.485 (3)	0.2802 (13)	0.5849 (9)	0.021 (5)*	
H21C	0.546 (3)	0.2208 (16)	0.5503 (10)	0.034 (7)*	
C22	0.62173 (15)	0.30815 (9)	0.47251 (5)	0.0181 (2)	
H22A	0.530 (2)	0.3126 (12)	0.4577 (8)	0.014 (5)*	
H22B	0.683 (2)	0.3415 (13)	0.4582 (8)	0.015 (5)*	
H22C	0.658 (3)	0.2589 (15)	0.4673 (10)	0.027 (6)*	

### Dibromidobis({(S)-2-[1-(dimethylamino)ethyl]phenyl}diphenylsilanol-κO)zinc(II) Atomic displacement parameters (Å^2^)

**Table:**

	*U*^11^	*U*^22^	*U*^33^	*U*^12^	*U*^13^	*U*^23^
Br1	0.01733 (5)	0.01215 (5)	0.02303 (6)	0.00417 (4)	0.00357 (5)	−0.00182 (4)
Zn1	0.01005 (7)	0.00799 (7)	0.00967 (7)	0.000	0.000	0.00028 (6)
Si1	0.01207 (13)	0.00994 (12)	0.00887 (12)	0.00211 (10)	−0.00017 (10)	−0.00023 (10)
O1	0.0139 (4)	0.0123 (3)	0.0096 (3)	0.0031 (3)	−0.0008 (3)	0.0001 (3)
N1	0.0131 (4)	0.0122 (4)	0.0114 (4)	0.0019 (3)	−0.0006 (3)	−0.0007 (3)
C1	0.0143 (5)	0.0160 (5)	0.0109 (4)	0.0030 (4)	0.0010 (4)	0.0011 (4)
C2	0.0154 (5)	0.0235 (5)	0.0140 (5)	−0.0002 (5)	0.0006 (4)	0.0004 (4)
C3	0.0145 (5)	0.0380 (8)	0.0177 (6)	−0.0018 (5)	−0.0002 (4)	0.0053 (5)
C4	0.0161 (6)	0.0437 (9)	0.0186 (6)	0.0061 (6)	0.0047 (5)	0.0063 (6)
C5	0.0254 (6)	0.0330 (7)	0.0155 (5)	0.0084 (6)	0.0081 (5)	0.0017 (6)
C6	0.0212 (5)	0.0216 (6)	0.0125 (4)	0.0034 (5)	0.0032 (4)	−0.0006 (4)
C7	0.0162 (5)	0.0117 (4)	0.0118 (5)	0.0016 (4)	−0.0012 (4)	−0.0003 (4)
C8	0.0217 (6)	0.0117 (4)	0.0154 (5)	0.0026 (4)	0.0005 (4)	−0.0003 (4)
C9	0.0301 (7)	0.0123 (5)	0.0186 (6)	−0.0015 (5)	0.0018 (5)	−0.0031 (4)
C10	0.0294 (7)	0.0181 (6)	0.0201 (6)	−0.0078 (5)	0.0002 (5)	−0.0028 (5)
C11	0.0206 (6)	0.0209 (6)	0.0264 (7)	−0.0041 (5)	−0.0014 (5)	−0.0021 (5)
C12	0.0171 (5)	0.0154 (5)	0.0204 (6)	0.0004 (4)	−0.0004 (4)	−0.0024 (4)
C13	0.0136 (4)	0.0118 (4)	0.0101 (4)	0.0013 (4)	−0.0013 (3)	0.0008 (3)
C14	0.0217 (6)	0.0160 (5)	0.0113 (4)	0.0044 (4)	−0.0013 (4)	0.0005 (4)
C15	0.0291 (7)	0.0207 (6)	0.0129 (5)	0.0065 (5)	−0.0030 (5)	0.0034 (4)
C16	0.0319 (7)	0.0208 (6)	0.0172 (5)	0.0106 (5)	−0.0042 (5)	0.0048 (5)
C17	0.0249 (6)	0.0163 (5)	0.0172 (5)	0.0086 (5)	−0.0033 (5)	0.0003 (4)
C18	0.0144 (5)	0.0119 (4)	0.0118 (4)	0.0028 (4)	−0.0017 (4)	0.0002 (3)
C19	0.0123 (4)	0.0143 (4)	0.0124 (4)	0.0035 (4)	−0.0007 (4)	−0.0011 (3)
C20	0.0149 (5)	0.0207 (5)	0.0158 (5)	−0.0015 (4)	0.0021 (4)	−0.0013 (4)
C21	0.0158 (5)	0.0141 (5)	0.0187 (5)	−0.0009 (4)	0.0002 (4)	−0.0006 (4)
C22	0.0188 (5)	0.0235 (6)	0.0120 (5)	0.0032 (5)	−0.0007 (4)	−0.0031 (4)

### Dibromidobis({(S)-2-[1-(dimethylamino)ethyl]phenyl}diphenylsilanol-κO)zinc(II) Geometric parameters (Å, º)

**Table:**

Br1—Zn1	2.3969 (2)	C10—H10	0.88 (3)
Zn1—O1^i^	1.9509 (9)	C10—C11	1.391 (2)
Zn1—O1	1.9509 (9)	C11—H11	0.95 (3)
Si1—O1	1.6135 (9)	C11—C12	1.392 (2)
Si1—C1	1.8802 (13)	C12—H12	0.89 (2)
Si1—C7	1.8819 (13)	C13—C14	1.4074 (17)
Si1—C13	1.8996 (12)	C13—C18	1.4114 (16)
N1—H1	0.95 (2)	C14—H14	0.92 (2)
N1—C19	1.5125 (17)	C14—C15	1.3933 (19)
N1—C21	1.4895 (18)	C15—H15	0.97 (2)
N1—C22	1.4897 (16)	C15—C16	1.386 (2)
C1—C2	1.4046 (19)	C16—H16	0.85 (3)
C1—C6	1.4042 (18)	C16—C17	1.392 (2)
C2—H2	0.94 (2)	C17—H17	0.98 (3)
C2—C3	1.393 (2)	C17—C18	1.3980 (18)
C3—H3	0.92 (3)	C18—C19	1.5267 (16)
C3—C4	1.390 (2)	C19—H19	0.92 (2)
C4—H4	0.92 (3)	C19—C20	1.5296 (19)
C4—C5	1.381 (3)	C20—H20A	1.01 (3)
C5—H5	1.01 (3)	C20—H20B	0.98 (2)
C5—C6	1.400 (2)	C20—H20C	0.89 (3)
C6—H6	0.94 (2)	C21—H21A	0.96 (2)
C7—C8	1.4043 (18)	C21—H21B	0.90 (2)
C7—C12	1.404 (2)	C21—H21C	0.95 (3)
C8—H8	0.95 (3)	C22—H22A	0.94 (2)
C8—C9	1.392 (2)	C22—H22B	0.90 (2)
C9—H9	0.96 (3)	C22—H22C	0.94 (3)
C9—C10	1.394 (2)		
			
Br1—Zn1—Br1^i^	114.035 (11)	C10—C11—H11	119.5 (16)
O1—Zn1—Br1^i^	108.75 (3)	C10—C11—C12	119.87 (15)
O1^i^—Zn1—Br1	108.75 (3)	C12—C11—H11	120.6 (16)
O1^i^—Zn1—Br1^i^	111.18 (3)	C7—C12—H12	120.5 (15)
O1—Zn1—Br1	111.18 (3)	C11—C12—C7	121.87 (14)
O1^i^—Zn1—O1	102.34 (6)	C11—C12—H12	117.6 (15)
O1—Si1—C1	109.27 (5)	C14—C13—Si1	112.61 (9)
O1—Si1—C7	114.35 (5)	C14—C13—C18	116.66 (11)
O1—Si1—C13	110.10 (5)	C18—C13—Si1	130.68 (9)
C1—Si1—C7	111.12 (6)	C13—C14—H14	117.9 (14)
C1—Si1—C13	106.39 (6)	C15—C14—C13	123.03 (12)
C7—Si1—C13	105.27 (5)	C15—C14—H14	119.1 (14)
Si1—O1—Zn1	137.64 (5)	C14—C15—H15	122.3 (13)
C19—N1—H1	102.5 (14)	C16—C15—C14	119.17 (12)
C21—N1—H1	109.3 (14)	C16—C15—H15	118.5 (13)
C21—N1—C19	112.50 (10)	C15—C16—H16	120.0 (18)
C21—N1—C22	109.17 (11)	C15—C16—C17	119.37 (13)
C22—N1—H1	111.2 (14)	C17—C16—H16	120.3 (18)
C22—N1—C19	112.10 (10)	C16—C17—H17	119.0 (15)
C2—C1—Si1	116.89 (9)	C16—C17—C18	121.48 (13)
C6—C1—Si1	125.89 (10)	C18—C17—H17	119.5 (15)
C6—C1—C2	117.21 (12)	C13—C18—C19	124.32 (10)
C1—C2—H2	117.9 (15)	C17—C18—C13	120.25 (11)
C3—C2—C1	121.52 (13)	C17—C18—C19	115.43 (11)
C3—C2—H2	120.6 (15)	N1—C19—C18	110.97 (10)
C2—C3—H3	117.2 (17)	N1—C19—H19	107.7 (14)
C4—C3—C2	120.01 (15)	N1—C19—C20	110.37 (10)
C4—C3—H3	122.8 (17)	C18—C19—H19	106.3 (14)
C3—C4—H4	119.0 (17)	C18—C19—C20	112.74 (10)
C5—C4—C3	119.80 (14)	C20—C19—H19	108.5 (14)
C5—C4—H4	121.0 (17)	C19—C20—H20A	108.0 (15)
C4—C5—H5	119.1 (16)	C19—C20—H20B	115.2 (13)
C4—C5—C6	120.17 (14)	C19—C20—H20C	113.2 (17)
C6—C5—H5	120.7 (16)	H20A—C20—H20B	104.9 (19)
C1—C6—H6	117.4 (15)	H20A—C20—H20C	107 (2)
C5—C6—C1	121.24 (14)	H20B—C20—H20C	108 (2)
C5—C6—H6	121.4 (14)	N1—C21—H21A	106.4 (15)
C8—C7—Si1	123.09 (10)	N1—C21—H21B	112.8 (15)
C12—C7—Si1	119.93 (10)	N1—C21—H21C	107.4 (17)
C12—C7—C8	116.98 (13)	H21A—C21—H21B	112 (2)
C7—C8—H8	120.6 (15)	H21A—C21—H21C	111 (2)
C9—C8—C7	121.68 (14)	H21B—C21—H21C	107 (2)
C9—C8—H8	117.7 (15)	N1—C22—H22A	107.7 (13)
C8—C9—H9	119.1 (17)	N1—C22—H22B	109.6 (14)
C8—C9—C10	119.99 (15)	N1—C22—H22C	110.8 (16)
C10—C9—H9	120.9 (17)	H22A—C22—H22B	110.8 (18)
C9—C10—H10	121.7 (18)	H22A—C22—H22C	110 (2)
C11—C10—C9	119.59 (15)	H22B—C22—H22C	108 (2)
C11—C10—H10	118.7 (18)		
			
Si1—C1—C2—C3	178.68 (12)	C7—Si1—C13—C18	−113.32 (12)
Si1—C1—C6—C5	179.58 (12)	C7—C8—C9—C10	0.5 (2)
Si1—C7—C8—C9	−179.84 (11)	C8—C7—C12—C11	−0.1 (2)
Si1—C7—C12—C11	179.22 (12)	C8—C9—C10—C11	0.3 (3)
Si1—C13—C14—C15	177.65 (12)	C9—C10—C11—C12	−0.9 (3)
Si1—C13—C18—C17	−175.57 (11)	C10—C11—C12—C7	0.8 (2)
Si1—C13—C18—C19	4.9 (2)	C12—C7—C8—C9	−0.6 (2)
O1—Si1—C1—C2	33.64 (11)	C13—Si1—O1—Zn1	158.45 (8)
O1—Si1—C1—C6	−147.67 (11)	C13—Si1—C1—C2	−85.19 (11)
O1—Si1—C7—C8	96.35 (12)	C13—Si1—C1—C6	93.50 (12)
O1—Si1—C7—C12	−82.91 (12)	C13—Si1—C7—C8	−142.69 (11)
O1—Si1—C13—C14	−166.90 (10)	C13—Si1—C7—C12	38.05 (13)
O1—Si1—C13—C18	10.39 (14)	C13—C14—C15—C16	−1.3 (2)
C1—Si1—O1—Zn1	41.96 (10)	C13—C18—C19—N1	−57.05 (15)
C1—Si1—C7—C8	−27.92 (13)	C13—C18—C19—C20	67.34 (16)
C1—Si1—C7—C12	152.82 (11)	C14—C13—C18—C17	1.63 (19)
C1—Si1—C13—C14	−48.61 (11)	C14—C13—C18—C19	−177.95 (12)
C1—Si1—C13—C18	128.68 (12)	C14—C15—C16—C17	1.1 (3)
C1—C2—C3—C4	1.6 (2)	C15—C16—C17—C18	0.4 (3)
C2—C1—C6—C5	−1.7 (2)	C16—C17—C18—C13	−1.9 (2)
C2—C3—C4—C5	−1.3 (3)	C16—C17—C18—C19	177.75 (15)
C3—C4—C5—C6	−0.6 (3)	C17—C18—C19—N1	123.36 (12)
C4—C5—C6—C1	2.1 (2)	C17—C18—C19—C20	−112.25 (13)
C6—C1—C2—C3	−0.1 (2)	C18—C13—C14—C15	−0.1 (2)
C7—Si1—O1—Zn1	−83.29 (10)	C21—N1—C19—C18	−47.13 (13)
C7—Si1—C1—C2	160.74 (10)	C21—N1—C19—C20	−172.85 (10)
C7—Si1—C1—C6	−20.57 (13)	C22—N1—C19—C18	−170.62 (10)
C7—Si1—C13—C14	69.39 (11)	C22—N1—C19—C20	63.66 (13)

Symmetry code: (i) *x*, −*y*+1, −*z*+1.

### Dibromidobis({(S)-2-[1-(dimethylamino)ethyl]phenyl}diphenylsilanol-κO)zinc(II) Hydrogen-bond geometry (Å, º)

**Table:**

*D*—H···*A*	*D*—H	H···*A*	*D*···*A*	*D*—H···*A*
C21—H21*A*···Br1^i^	0.96 (2)	3.10 (2)	3.8552 (14)	137.0 (18)
C22—H22*A*···Br1^i^	0.94 (2)	2.95 (2)	3.7373 (14)	142.1 (17)
C19—H19···Br1^ii^	0.92 (2)	3.09 (2)	3.8944 (11)	146.9 (17)
C21—H21*C*···Br1^ii^	0.95 (3)	2.86 (3)	3.7615 (14)	159 (2)
C22—H22*B*···*Cg*(C7–12)^i^	0.90 (2)	2.84 (2)	3.4972 (15)	131 (2)
C15—H15···*Cg*(1–6)^iii^	0.97 (2)	3.11 (2)	3.7553 (16)	125 (2)

Symmetry codes: (i) *x*, −*y*+1, −*z*+1; (ii) *x*+1/2, *y*−1/2, *z*; (iii) −*x*+1, *y*, −*z*+3/2.
